# Perception of attractiveness of missing maxillary lateral incisors replaced by canines

**DOI:** 10.1590/2177-6709.23.5.065-074.oar

**Published:** 2018

**Authors:** Ricardo Alves de Souza, Girlaine Nunes Alves, Juliana Macêdo de Mattos, Raildo da Silva Coqueiro, Matheus Melo Pithon, João Batista de Paiva

**Affiliations:** 1 Universidade de São Paulo, Faculdade de Odontologia, Departamento de Ortodontia (São Paulo/SP, Brazil). Universidade de São Paulo Universidade de São Paulo Faculdade de Odontologia Departamento de Ortodontia São PauloSP Brazil; 2 Faculdade de Tecnologia e Ciências, Departamento de Ortodontia (Jequié/BA, Brazil). Faculdade de Tecnologia e Ciências Departamento de Ortodontia JequiéBA Brazil; 3 Universidade Estadual do Sudoeste da Bahia, Departamento de Saúde (Jequié/BA, Brazil). Universidade Estadual do Sudoeste da Bahia Universidade Estadual do Sudoeste da Bahia Departamento de Saúde JequiéBA Brazil; 4 Universidade Estadual do Sudoeste da Bahia, Departamento de Ortodontia (Jequié/BA, Brazil). Universidade Estadual do Sudoeste da Bahia Universidade Estadual do Sudoeste da Bahia Departamento de Ortodontia JequiéBA Brazil

**Keywords:** Anodontia, Esthetics, Space closure, Visual perception

## Abstract

**Objective:**

The aim of this study was to evaluate the degree of perception of attractiveness of the smile among dentists, dental students, and lay persons in cases of agenesis of the maxillary lateral incisors replaced by canines for space closure.

**Methods:**

A smiling front view extraoral photograph of a 20-year-old woman was digitally altered simulating agenesis and its treatment, by means of: repositioning, reshaping or bleaching the canine, and gingival contour. A questionnaire was distributed to individuals of the three groups (*n*= 150), with a view to evaluating their degree of esthetic perception. An attractiveness scale was also used, with ‘0’ representing unattractive and ‘10’, very attractive.

**Results:**

In the comparative evaluation among all the photographs, the original image obtained the highest level of acceptance. Photograph ‘i’ (agenesis of both lateral incisors treated with reposition and reshaping of the canines) was ranked as the least attractive by the dentists, whereas the student and lay persons ranked photograph ‘f’ (agenesis of both lateral incisors treated with reposition of the canines, gingival contour, bleaching and reshaping) as the worst.

**Conclusion:**

The methods of treatment most accepted among the dentists and students were those that involved changes in the gingival contour, whereas among lay persons, they were those that involved only reshaping.

## RESUMO

**Objetivo:**

o objetivo do presente estudo foi avaliar o grau de percepção da atratividade do sorriso por dentistas, estudantes de Odontologia e leigos, em casos de agenesia dos incisivos laterais superiores substituídos por caninos, para fechamento do espaço.

**Métodos:**

uma fotografia extrabucal frontal do sorriso de uma mulher com 20 anos de idade foi alterada simulando agenesia e seu tratamento por meio de: reposicionamento do canino, remodelação, clareamento ou contorno gengival. Um questionário foi distribuído aos indivíduos dos três grupos (*n*= 150), com o objetivo de avaliar o grau de percepção estética. Uma escala de atratividade também foi usada, com ‘0’ representando pouco atraente e ‘10’, muito atraente.

**Resultados:**

na avaliação comparativa entre as fotografias, a imagem original obteve o maior nível de aceitação. A fotografia “i” (agenesia de ambos os incisivos laterais tratada com reposição e remodelação dos caninos) foi classificada como a menos atraente pelos dentistas, enquanto os estudantes e os leigos classificaram a “f” (agenesia de ambos os incisivos laterais tratada com reposição dos caninos, contorno gengival, clareamento e remodelação) como a pior.

**Conclusão:**

os métodos de tratamento mais aceitos entre dentistas e estudantes foram aqueles que envolviam mudanças no contorno gengival, enquanto entre os leigos foram aqueles que envolviam apenas remodelação.

## INTRODUCTION

Agenesis of the teeth is the most common craniofacial development anomaly in humans. It represents an anomaly in number characterized by the absence of one or more teeth, which may be linked to genetic or environmental factors^1^^-^^5^ or associated with syndromes,^2^^,^^4^ with the former being related to the larger portion of cases. In fact, the expression of more than 200 genes is responsible for tooth development, so a mutation in any of these may hinder this process.^4^

Because it is a common problem in contemporary man, over the last few years various studies have sought to describe the prevalence of hypodontia in different groups. It is relatively frequent in permanent dentition, with an incidence between 0.3% and 11.3% in different populations, excluding the third molars, and an even higher value if these teeth were considered.^5^ The maxillary lateral incisors, depending on the ethnic group, may present the highest^3^^,^^6^ or second highest incidence.^7^^,^^8^^,^^9^

There are two basic treatment options for congenitally missing maxillary lateral incisors: creating adequate space, placing maxillary canine into its natural position and subsequently replacing the missing lateral incisor with prostheses; or closing the space available in the dental arch, providing contact of the central incisor with the canine, and afterwards proceeding with reshaping of the canine, transforming it into a lateral incisor and the first premolar into the position of the canine. The space closure alternative could be associated with tooth remodeling and restorations of the canine.^4^^,^^5^^,^^9^^-^^13^

Clinically, absence of the lateral incisor may generate some esthetic, periodontal and functional problems. The harmony of the smile is compromised by these alterations, as they generate diastemas and promote dimensional alterations by changing the shape, size and the proportion of height/width of the teeth, resulting in unattractive facial appearance.^4^ A consensus about the best treatment in terms of functional and esthetic needs of the patient was not achieved, and it involves not only orthodontists but also general dentists, and the perception of patients is also important.^4^^,^^11^^,^^12^

Starting with the principle that perception of dental esthetics is different among the groups, the aim of this study was to quantitatively evaluate the attractiveness of photographs of the smile in a patient with agenesis of the maxillary lateral incisors that were replaced by canines, followed by different changes as regards the number of missing maxillary lateral incisors, incisor morphology, and gingival margin height and color.

## MATERIAL AND METHODS 

This study was approved by the Ethics Committee on Human Research of *Universidade Estadual do Sudoeste da Bahia* (CCS/UESB, CEP/CAAE #17333113.1.0000.0055).

To conduct this study, a front view extraoral photograph of a 20-year-old woman patient with normal occlusion was used. The photograph used was captured with a digital photographic camera (10 megapixels; Canon XTI Rebel, Japan), resulting in an image in which the inferior third of the patient’s face, including the lips, gingival tissue and teeth, could be clearly visualized.

The original photograph was manipulated with the aid of the Adobe Photoshop CS3 software (Adobe Systems Inc, San Francisco, CA, USA) however, maintaining the mandibular arch without any modification.

The alterations in the photograph were made in the anterior region of maxillary arch, with various compositions of different sizes and proportions of height and width of the teeth. Changes were made with the intention of simulating repositioning of the canine in the left, right or both sides, in an individualized manner, in the place of the lateral incisor. 

**Figure 1:**
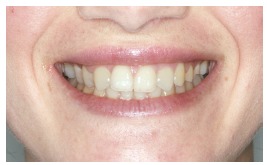
Repositioning the crown and gingival contour.

**Figure 2:**
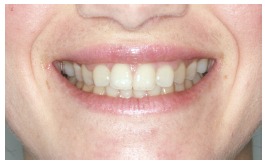
Repositioning, bleaching reshaping of the crown, and gingival contour.

**Figure 3:**
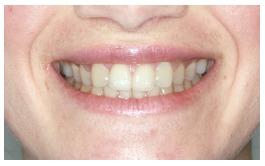
Repositioning and reshaping of the crown.

**Figure 4:**
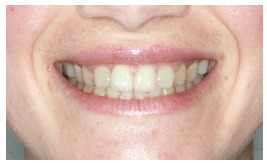
Repositioning, reshaping of the crown, and gingival contour.

**Figure 5:**
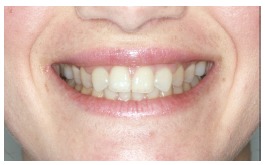
Repositioning, bleaching the crown, and gingival contour.

**Figure 6:**
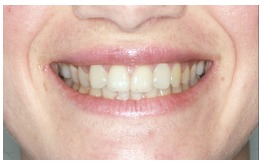
Repositioning, reshaping and bleaching the crown.

**Figure 7:**
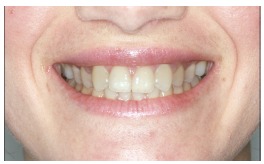
Repositioning the crown.

**Figure 8:**
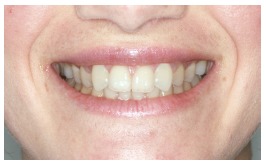
Repositioning and bleaching the crown.

The groups of images were divided according to the suggested treatment, consisting of: repositioning the crown and gingival contour (Fig 1); repositioning, bleaching and reshaping of the crown, and gingival contour (Fig 2); repositioning and reshaping of the crown (Fig 3); repositioning and reshaping of the crown, and gingival contour (Fig 4); repositioning and bleaching the crown, and gingival contour (Fig 5); repositioning, reshaping and bleaching the crown (Fig 6); repositioning the crown (Fig 7); and repositioning and bleaching the crown (Fig 8).

**Figure 9:**
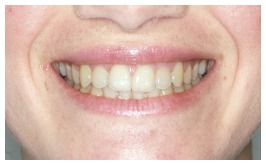
Original photograph.

Twenty-five different photographs were obtained, thus making up the following groups, according to where the changes were made: original photograph (Fig 9) represented by the letter ‘a’; photographs with changes in the right side (b, e, h, k, n, q, t, w); photographs with changes in both sides (c, f, i, l, o, r, u, x); and photographs with changes in the left side (d, g, j, m, p, s, v, y). The images were randomly printed on photographic paper and attached to a questionnaire with the aid of a Visual Analog Scale (VAS) and evenly distributed to lay persons, dentists and dental students (*n*= 150). In this scale, score ‘0’ corresponded to a unattractive image; ‘5’, to an attractive image; and ‘10’, to a very attractive image. All the evaluators were advised not to compare the images of different sheets. In a second moment the images were compared to each other and questions were asked if it was possible to see differences between them and afterwards put them in an order of better aesthetics. The evaluation time interval for each image was limited to 60 seconds. 

The 150 people who answered the questionnaire were equally divided among dental students who were between the 5th and 9th semester, dental surgeons with at least one year of graduation and no specialization, and lay persons who were students of other courses in the university. The age of the whole sample ranged between 22 and 40 years old and showed similar socioeconomic status. 

A sample calculation was performed to determine how many people would respond to the questionnaire. The following parameters were considered for the sample calculations: test power of 80% (β = 0.20) and error of 5% (α = 0.05). The sample calculations were performed in BioEstat v. 5.3 ((Instituto Mamirauá, Brazil), and the minimum amount was increased by 15%.

### Statistical procedures

The frequencies of the answers given by the dentists, dental students and laypersons were compared by means of the exact Fisher test. The normality of the scores attributed to each photograph was verified by means of the Kolmogorov-Smirnov test and by homogeneity variances of the Levene test. Since all the data had normality assumptions and/or homogeneity variances violated, the non-parametric statistic was performed by Kruskal-Wallis test, with the comparisons between pairs being tested by the Mann-Whitney test. The means of grades awarded to each photograph were calculated in each group in order to determine the Spearman correlation coefficients, to evaluate the similarity between the perceptions of the dental professionals, dental students and laypersons. The level of significance adopted was 5% (α = 0.05). The data were tabulated and analyzed in the statistical program BioEstat (v. 5.3, Belém/PA, Brazil).

## RESULTS

Table 1 shows the demographic data of the study participants. Of the 150 individuals, 53.3% were men and the majority (82.0%) were in the age group ≤ 30 years.

**Table 1:** Demographic data of study evaluators.

	Dental surgeon	Dental students	Lay persons
	(n = 50)	(n = 50)	(n = 50)
Characteristics / Sex			
Male	26 (52.0%)	18 (36.0%)	36 (72.0%)
Female	24 (48.0%)	32 (64.0%)	14 (28.0%)
Age ≤ 30 years	25 (50.0%)	48 (96.0%)	50 (100%)
Age > 30 years	25 (50.0%)	2 (4.0%)	0 (0.0%)

For all the images presented, there were significant differences between the three groups of evaluators in the choice of the best photograph.

Table 2 shows the research participants’ and the group of evaluators’ perception according to the photographs they liked most. For all the images presented, there was significant differences between the three groups of evaluators in the choice of the best photograph, with the frequency of evaluators who opted for photograph ‘a’ being higher in the group of dentists, followed by the group of dental students.

**Table 2:** Perception of evaluators as regards the best photography.

Figures	Dental surgeons	Dental students	Lay persons	p-value
Fig. 1*				
a ^o^	48 (96.0%)	42 (84.0%)	16 (35.6%)	< 0.001
b ^AR, GC^	2 (4.0%)	1 (2.0%)	18 (40.0%)	
c ^ARL, GC^	0 (0.0%)	2 (4.0%)	4 (8.9%)	
d ^AL, GC^	0 (0.0%)	5 (10.0%)	7 (15.6%)	
Fig. 2*				
a ^o^	49 (98.0%)	45 (90.0%)	33 (70.2%)	<0.001
e ^AR, B, GC, R^	1 (2.0%)	0 (0.0%)	10 (21.3%)	
f ^ARL, B, GC, R^	0 (0.0%)	4 (8.0%)	2 (4.3%)	
g ^AL, B, GC, R^	0 (0.0%)	1 (2.0%)	2 (4.3%)	
Fig. 3*				
a ^o^	49 (98.0%)	42 (84.0%)	36 (73.5%)	0.005
h ^AR, R^	1 (2.0%)	3 (6.0%)	9 (18.4%)	
i ^ARL, R^	0 (0.0%)	2 (4.0%)	3 (6.1%)	
j ^AL, R^	0 (0.0%)	3 (6.0%)	1 (2.0%)	
Fig. 4*				
a ^o^	48 (96.0%)	44 (88.0%)	34 (69.4%)	< 0.001
k ^AR, GC, R^	1 (2.0%)	4 (8.0%)	14 (28.6%)	
l ^ARL, GC, R^	1 (2.0%)	1 (2.0%)	0 (0.0%)	
m ^AL, GC, R^	0 (0.0%)	1 (2.0%)	1 (2.0%)	
Fig. 5*				
a ^o^	48 (96.0%)	44 (88.0%)	37 (75.5%)	0.031
n ^AR, B, GC^	1 (2.0%)	3 (6.0%)	9 (18.4%)	
o ^ARL, B, GC^	1 (2.0%)	1 (2.0%)	2 (4.1%)	
p ^AL, B, GC^	0 (0.0%)	2 (4.0%)	1 (2.0%)	
Fig. 6*				
a ^o^	48 (96.0%)	40 (81.6%)	26 (55.3%)	< 0.001
q ^AR, B, R^	1 (2.0%)	6 (12.2%)	13 (27.7%)	
r ^ARL, B, R^	1 (2.0%)	2 (4.1%)	7 (14.9%)	
s ^AL, B, R^	0 (0.0%)	1 (2.0%)	1 (2.1%)	
Fig. 7*				
a ^o^	49 (98.0%)	41 (82.0%)	26 (54.2%)	< 0.001
t ^AR^	0 (0.0%)	4 (8.0%)	11 (22.9%)	
u ^ARL^	1 (2.0%)	4 (8.0%)	7 (14.6%)	
v ^AL^	0 (0.0%)	1 (2.0%)	4 (8.3%)	
Fig. 8*				
a ^o^	49 (98.0%)	37 (80.4%)	25 (52.1%)	< 0.001
w ^AR, B^	0 (0.0%)	2 (4.3%)	11 (22.9%)	
x ^ARL, B^	1 (2.0%)	4 (8.7%)	8 (16.7%)	
y ^AL, B^	0 (0.0%)	3 (6.5%)	4 (8.3%)	

*The participants who did not note any differences in the photographs were not included. ^O^ Original image. ^AR^ Absence of unit 12.

A ^RL^ Absence of units 12 and 22. ^AL^ Absence of unit 22. ^B^ Bleaching. ^GC^ Gingival Contour. ^R^ Reanatomization.

Table 3 shows the research participants’ and the group of evaluators’ perception according to the photographs they liked least. For images 1, 3, 4 and 8 there was significant difference between the three groups of evaluators in the choice of the worst photograph.

**Table 3:** Perception of evaluators as regards the worst photography.

Figures	Dental surgeons	Dental students	Lay persons	p-value
Fig. 1*				
a ^o^	0 (0.0%)	1 (2.0%)	5 (11.1%)	0.020
b ^AR, GC^	8 (16.0%)	12 (24.0%)	6 (13.3%)	
c ^ARL, GC^	12 (24.0%)	19 (38.0%)	9 (20.0%)	
d ^AL, GC^	30 (60.0%)	18 (36.0%)	25 (55.6%)	
Fig. 2*				
a ^o^	1 (2.0%)	1 (2.0%)	3 (6.4%)	0.361
e ^AR, B, GC, R^	5 (10.0%)	7 (14.0%)	3 (6.4%)	
f ^ARL, B, GC, R^	21 (42.0%)	28 (56.0%)	21 (44.7%)	
g ^AL, B, GC, R^	23 (46.0%)	14 (28.0%)	20 (42.6%)	
Fig. 3*				
a ^o^	0 (0.0%)	6 (12.0%)	4 (8.2%)	< 0.001
h ^AR, R^	3 (6.0%)	2 (4.0%)	3 (6.1%)	
i ^ARL, R^	22 (44.0%)	34 (68.0%)	15 (30.6%)	
j ^AL, R^	25 (50.0%)	8 (16.0%)	27 (55.1%)	
Fig. 4*				
a ^o^	1 (2.0%)	1 (2.0%)	6 (12.2%)	0.004
k ^AR, GC, R^	11 (22.0%)	5 (10.0%)	2 (4.1%)	
l ^ARL, GC, R^	16 (32.0%)	30 (60.0%)	25 (51.0%)	
m ^AL, GC, R^	22 (44.0%)	14 (28.0%)	16 (32.7%)	
Fig. 5*				
a ^o^	1 (2.0%)	2 (4.0%)	4 (8.2%)	0.085
n ^AR, B, GC^	4 (8.0%)	3 (6.0%)	2 (4.1%)	
o ^ARL, B, GC^	11 (22.0%)	24 (48.0%)	16 (32.7%)	
p ^AL, B, GC^	34 (68.0%)	21 (42.0%)	27 (55.1%)	
Fig. 6*				
a ^o^	1 (2.0%)	1 (2.0%)	3 (6.4%)	0.665
q ^AR, B, R^	6 (12.0%)	5 (10.2%)	4 (8.5%)	
r ^ARL, B, R^	20 (40.0%)	27 (55.1%)	21 (44.7%)	
s ^AL, B, R^	23 (46.0%)	16 (32.7%)	19 (40.4%)	
Fig. 7*				
a ^o^	1 (2.0%)	1 (2.0%)	6 (12.5%)	0.199
t ^AR^	7 (14.0%)	5 (10.0%)	5 (10.4%)	
u ^ARL^	18 (36.0%)	26 (52.0%)	20 (41.7%)	
v ^AL^	24 (48.0%)	18 (36.0%)	17 (35.4%)	
Fig. 8*				
a ^o^	1 (2.0%)	1 (2.0%)	3 (6.3%)	0.009
w ^AR, B^	8 (16.0%)	3 (6.5%)	4 (8.3%)	
x ^ARL, B^	13 (26.0%)	29 (63.0%)	19 (39.6%)	
y ^AL, B^	28 (56.0%)	13 (28.3%)	22 (45.8%)	

*The participants who did not note any differences in the photographs were not included. ^O^ Original image. ^AR^ Absence of unit 12.

A ^RL^ Absence of units 12 and 22. ^AL^ Absence of unit 22. ^B^ Bleaching. ^GC^ Gingival Contour. ^R^ Reanatomization.

Table 4 shows that almost all the evaluators of the study were able to note differences between the images presented in the photographs; but only for the image with repositioning of the crown and gingival contour (Fig 1) there was a significant difference (*p*= 0.011) between the groups of evaluators, with dental surgeons (100%) and dental students (100%) being more capable to notice differences than lay people (90%).

**Table 4:** Median scores (interquartile range) of the photographs.

Photograph	Dental surgeons	Dental students	Lay persons	p-value
Image a ^O^	8.00 (1.00)^a^	8.00 (2.00)^a^	5.00 (3.00)^b^	< 0.001
Image b ^AR, GC^	6.00 (2.00)^a^	5.50 (3.00)^a^	5.00 (2.30)^b^	0.006
Image c ^ARL, GC^	5.00 (1.00)^a^	4.00 (2.90)^b^	4.50 (3.00)^b^	0.010
Image d ^AL, GC^	5.00 (2.00)	5.00 (2.00)	5.00 (2.00)	0.377
Image e ^AR, B, GC, R^	4.25 (2.00)	4.75 (3.00)	5.00 (2.60)	0.838
Image f ^ARL, B, GC, R^	4.00 (2.50)^a^	3.00 (2.50)^b^	4.00 (3.00)^ab^	0.020
Image g ^AL, B, GC,R^	4.00 (2.00)	4.00 (2.10)	4.00 (2.50)	0.884
Image h ^AR, R^	5.00 (2.00)	5.00 (3.00)	4.00 (2.50)	0.078
Image i ^ARL, R^	4.00 (2.00)^a^	3.00 (2.50)^b^	4.00 (2.10)^a^	0.026
Image j ^AL, R^	4.75 (2.50)	4.25 (2.00)	5.00 (2.50)	0.608
Image k ^AR, GC, R^	6.75 (2.00)	5.50 (1.60)	5.50 (3.00)	0.060
Image l ^ARL, GC, R^	6.00 (3.50)	4.25 (2.70)	5.50 (3.00)	0.115
Image m ^AL, GC, R^	6.00 (2.50)^a^	5.50 (2.50)^b^	5.25 (2.10)^b^	0.004
Image n ^AR, B, GC^	5.00 (2.00)	5.00 (4.00)	5.15 (2.00)	0.964
Image o ^ARL, B, GC^	6.00 (1.30)	5.25 (2.80)	5.50 (3.50)	0.551
Image p ^AL, B, GC^	5.00 (3.00)	5.00 (2.10)	5.00 (2.00)	0.333
Image q ^AR, B, R^	5.50 (2.00)	5.25 (2.80)	5.00 (2.00)	0.061
Image r ^ARL, B, R^	5.50 (3.00)	4.00 (3.00)	4.80 (3.00)	0.154
Image s ^AL, B, R^	5.50 (1.00)	5.00 (2.60)	5.00 (3.50)	0.368
Image t ^AR^	6.00 (2.00)^a^	6.00 (2.30)^b^	5.15 (2.50)^b^	0.012
Image u ^ARL^	6.00 (2.00)^a^	5.00 (3.00)^b^	5.25 (2.50)^ab^	0.017
Image v ^AL^	6.00 (1.60)^a^	5.00 (2.00)^b^	5.25 (2.00)^b^	0.017
Image w ^AR, B^	7.00 (1.80)^a^	6.00 (2.80)^b^	5.00 (2.60)^b^	< 0.001
Image x ^ARL, B^	7.00 (2.00)^a^	6.00 (2.60)^b^	5.50 (2.60)^b^	0.005
Image y ^AL, B^	7.00 (3.00)^a^	7.00 (3.00)^a^	5.70 (2.70)^b^	< 0.001

* The scores of grades were compared by means of the Kruskal-Wallis test.

a ^,b^ Values with different superscript letters were significantly different (Mann-Whitney test).

A ^R^ Absence of unit 12. ^ARL^ Absence of units 12 and 22. ^AL^ Absence of unit 22. ^B^ Bleaching. ^GC^ Gingival contour. ^R^ Reanatomization.

### Esthetic scores

The means of the scores for each photograph are shown in Table 4. Photograph ‘a’ was ranked as the most attractive by the dentists and dental students, while the lay persons scored ‘l’ as the best photograph. Photograph ‘i’ was ranked as the least attractive by the dentists, while the dental students and lay persons scored ‘f’ as the worst photograph. The scores of photographs showed significant differences between the groups. Comparisons between pairs showed that: dentists and dental students awarded better scores to photographs ‘a’, ‘b’, ‘m’ and ‘y’, compared with the lay persons; dentists gave better scores to photographs ‘c’, ‘t’, ‘v’, ‘w’ and ‘x’, compared with dental students and lay persons; dentists gave better scores to photographs ‘f’, ‘i’ and ‘u’, compared with dental students but not in comparison with lay persons. 

## DISCUSSION 

The development of teeth is a complex process, composed of a network of mechanisms and characterized by a series of morphological stages and physiological reactions.^14^ The singularity of this process is evident by the occurrence of one and the same chain of events producing all the teeth, although these have different forms and functions.^15^ Changes in this sequence of events may cause several disturbances in odontogenesis, such as change in the shape, size, structure or number of teeth. Among these problems, hypodontia is outstanding because it presents higher prevalence in comparison with all the above-mentioned disturbances.

The management of patients with agenesis of the maxillary lateral incisors must take into consideration various important questions that involve the amount of space, patient’s age, type of malocclusion and state of the adjacent teeth.^9^ The condition is more common bilaterally than unilaterally, and may originate various problems, such as unpleasant spacing between the anterior teeth and rotation of the central incisors and canines.^5^^,^^16^

Among the treatment options, space closure with reposition of the canine in the place of the missing lateral incisor is the best choice.^17^^,^^18^ However, some characteristics of the canines must be changed, so that these teeth have an esthetic appearance closer to those of the missing lateral incisors. These characteristics are: shape of the crown, gingival contour and color of these teeth that must be taken into consideration, as they lead to difficulty in obtaining an acceptable esthetic result.^19^

Based on this premise, the authors’ proposal in the present study was to evaluate the degree of perception of the attractiveness of the smile among dentists, dental students and lay persons in cases of agenesis of the lateral incisors and closing the space (resulting from this anomaly) with the canines, by means of alterations in photographs. Clinical situations were simulated, such as alteration a canine color with a view to minimizing some of the anatomic differences between canines and maxillary lateral incisors.

By means of using an image manipulation software (Adobe Photoshop CS3, Adobe Systems Inc, San Francisco, CA, USA), alterations were made in a front view photograph of a patient with normal occlusion and all the teeth. After obtaining the manipulated photographs, an album was mounted and attached to a questionnaire that was distributed among individuals of the three groups. The methodology of this researched is based on previous studies existent in the literature that modified an original photo to evaluate the esthetic perception of individuals, with a range of possible clinical situations.^20^^,^^21^

The 25 images were individually printed and evaluated as regards attractiveness, with the help of a visual analog scale (VAS). This method consists of using a scale graded from 0 to 10, where ‘0’ represents “hardly attractive” and ‘10’, “very attractive”. The results indicated that all the dental students and dentists observed differences between the images in an immediate viewing. However, in the group of lay persons, this percentage was lower when compared with the other two groups, presenting statistically significant difference. This fact corroborates data obtained in other researches, that professionals have an esthetic perception of the smile similar to that of dental students^22^^,^^23^ and differing from that of lay persons.^24^

The original photograph (a) had greater acceptance among all the images evaluated by all the groups, except in image ‘b’ (repositioning the crown and gingival contour in the canine of the right side), which was chosen by the lay individuals. Although there is agreement among the different professional groups as regards this photograph, it is necessary to point out that there was statistical difference with respect to the percentage. As regards a lower level of acceptance, the authors observed that the highest percentages of all the images were obtained by the alterations in the left side (d, g, j, m, p, s, y) and in both sides (c, f, i, l, o, r, u, x). Image ‘l’ (repositioning, gingival contour and reshaping of both maxillary canines) received the highest scores from the lay persons. Image ‘i’ (repositioning and reshaping) was the photograph that obtained the lowest mean score among the professionals, and ‘f’ (repositioning, bleaching, gingival contour and reshaping of the canines on both sides) among the lay persons and dental students.

However, the fact that draws attention in the group of dental students was that in all the images, the least attractive photograph was always the one with alterations in both sides. In the group of dentists this choice was always the image with alterations in the left side, whereas the group of lay persons’ choice alternated between the two previous options. 

In fifteen of the twenty-five images, the group made up of lay persons attributed the lowest mean scores in comparison with the other two groups. These results are in disagreement with those obtained in previous researches, in which esthetic perception was evaluated in different groups of professionals, and affirmed that lay persons are less strict, shown by attributing the highest mean scores in comparison with other professional groups.^12^^,^^25^

Research has shown that tooth color is of primary importance for esthetic satisfaction with treatment outcomes for laypersons.^13^ Studies underlined the importance of teeth color in the perception of smile, in which simulation with bleaching and periodontal contour was ranked more acceptable.^11^ But in the present study the least acceptable among all the groups were those in which bleaching was performed. This may have occurred because digital improvement of a natural clinical situation bears the risk of creating artificial looks, which may be less appealing than the natural image. In fact, the digitally bleached canines appear too opaque and lack the natural color gradient from the incisal edge to the gingival margin. It is possible that this has negatively influenced the negative rating for the bleached canines. In reality, patients (laypersons) are usually very satisfied after bleaching procedures for mesialized canines which substitute missing lateral incisors.

A fact that draws attention is that the images ‘t’, ‘u’ and ‘v’, in which there is absence of the lateral incisors without any type of treatment, were scored as attractive. Whereas image ‘f’, in which there is agenesis of both maxillary lateral incisors, with all the forms of treatment was considered the least attractive among the group of dentists and dental students. These results raise some questions: when there is space closure with repositioning of canines, is it necessary to reshape these teeth to give them an aspect similar to that of the missing lateral incisors? If lay persons recognize images simulating agenesis without any treatment as being attractive, why should dentists treat them? 

In most situations, the changes were noted. Only for repositioning of the crown and gingival contour images (Fig 1) there were significant differences between the groups of evaluators, with dental surgeons and dental students being more able to notice the differences than lay people. This shows that depending on the treatment performed on lay people, they can not see the treatment in the photographs. The bleaching and reshaping of the crown were treatments that were always perceived by all participants.

Available means of rehabilitation has its own advantages, disadvantages, indications, and limitations.^4^ These interventions for canine replacement promote changes that often are not favorable to the health of the canine tooth, in addition to bringing financial costs to the patient. Aggregating to clinical practice the knowledge obtained in studies of the perception of esthetic impact of diverse oral conditions is shown to be a fact of extreme importance. With respect to agenesis of maxillary lateral incisors, the use of the results of this research as subsidy for decision making in clinical planning allows the production of an attractive and pleasant smile, in which there is a harmonious relationship between the esthetics of facial structures. Therefore, the authors emphasize the need for respecting the patient’s opinion to achieve an individualized smile. Clinicians should consider the patient needs and preferences before choosing the treatment.

The discoveries of this study with this sample can not be generalized to the entire population. The aesthetics is subjective and varies depending of the region and culture, in a way that future researches should be done in different populations. 

## CONCLUSION

By conducting this study, the authors could infer that:

a) The clinician should choose the treatment taking into account patient’s preferences.
b) In general, none of the treatments evaluated had an acceptability similar to that obtained by the original image.
c) In the majority of situations, the lay persons were more critical in their evaluations, awarding lower scores than the dentists and dental students.
d) The methods of treatment most accepted among the dentists and dental students were those that involved changes in the gingival contour, whereas among lay persons, they were those that involved only reshaping. The least acceptable among all the groups were those in which bleaching was performed.
e) Similar studies should be carried out in different cultures.
